# Transport Properties of Natural and Artificial Smart Fabrics Impregnated by Graphite Nanomaterial Stacks

**DOI:** 10.3390/nano11041018

**Published:** 2021-04-16

**Authors:** Carola Esposito Corcione, Francesca Ferrari, Raffaella Striani, Antonio Greco

**Affiliations:** Dipartimento di Ingegneria dell’Innovazione, Università del Salento, 73100 Lecce, Italy; francesca.ferrari@unisalento.it (F.F.); raffaella.striani@unisalento.it (R.S.); antonio.greco@unisalento.it (A.G.)

**Keywords:** graphite nanomaterial stacks, electrical conductivity, thermal conductivity, textiles

## Abstract

In this work, we studied the transport properties (thermal and electrical conductivity) of smart fabric materials treated with graphite nanomaterial stacks–acetone suspensions. An innovative and easy method to produce graphite nanomaterial stacks–acetone-based formulations, starting from a low-cost expandable graphite, is proposed. An original, economical, fast, and easy method to increase the thermal and electrical conductivity of textile materials was also employed for the first time. The proposed method allows the impregnation of smart fabric materials, avoiding pre-coating of the fibers, thus reducing costs and processing time, while obtaining a great increase in the transport properties. Two kinds of textiles, cotton and Lycra^®^, were selected as they represent the most used natural and artificial fabrics, respectively. The impact of the dimensions of the produced graphite nanomaterial stacks–acetone-based suspensions on both the uniformity of the treatment and the transport properties of the selected textile materials was accurately evaluated using several experimental techniques. An empirical relationship between the two transport properties was also successfully identified. Finally, several theoretical models were applied to predict the transport properties of the developed smart fabric materials, evidencing a good agreement with the experimental data.

## 1. Introduction

Graphene is an atomically thick, two-dimensional (2D) sheet composed of sp2 carbon atoms arranged in a honeycomb structure. It could be considered the building block of all other graphitic carbon allotropes of different dimensionalities [1]. Nanocomposites reinforced with graphene platelets present improved electrical and thermal conductivity, as do nanocomposites reinforced with clay platelets, characterized by improved strength, modulus, heat distortion temperature, and barrier properties [2,3,4,5,6,7,8,9,10,11,12]. A great advantage of the graphite nanoplatelets from an economic point of view is that they are about 500 times less expensive than carbon nanotubes [13,14]. Furthermore, graphite platelets can be exfoliated and compounded in conventional processing routes in contrast with nanotube-based composites, which require the development of specific processing techniques able to control their dispersion, waviness, and alignment. For this reason, the graphite nanoplatelets represent a potential alternative to carbon nanotubes with regard to cost and improved target properties [15]. A wide range of scientific works report the synthesis of expandable graphite (GIC) as well as graphene nanosheets [16,17,18,19,20,21,22]. Conventionally, natural graphite (NG) is first converted to intercalated or expandable graphite through chemical oxidation in the presence of H_2_SO_4_ or HNO_3_. Expanded graphite (EG) is then obtained by expansion and exfoliation of GIC by rapid heating in a furnace above 600 °C. Several studies have been performed on EG-reinforced conductive polymer nanocomposites [22,23,24,25,26,27,28,29,30]. So far, investigations have been carried out on thermoplastic matrices, such as polystyrene [22], poly(methyl methacrylate) [23,24], nylon-6 [26], and polypropylene [27] for the production of nanocomposites via in situ polymerization or solution compounding. However, there is limited literature on the development of graphite nanomaterial stacks-based dispersions for the impregnation of smart textile materials with special focus on the improvement of electrical and heat transport properties. In recent years, graphite nanomaterials have attracted attention in the textile field aimed at developing graphite-nanomaterial-modified fibers, exploiting their several functional properties. In particular, cotton fibers coated with graphene demonstrated great potential with various applications, especially as smart textiles, protective clothing, and sensors [31]. A large increase in the performance of the functionalized fabric can only be obtained with a good connection between the fibers and the graphene stacks [32,33,34]. This is possible through chemical bonding with its oxidized functionalized derivative, i.e., graphene oxide (GO). The oxygen-bearing polar functional groups, such as carboxyl, carbonyl, hydroxyl, and epoxide, allow GO to be homogeneously dispersed in water by forming chemical bonds with the functional groups on the fabric surface. Coupling and crosslinking agents are often used to fortify the bonding between GO and fibers. Several methods to coat graphene on cotton fibers were recently developed. For example, Ag and Cu nanoparticles were incorporated into reduced GO (RGO), and cotton fabrics were successfully coated with modified RGO nanoparticles using 3-glycidyloxypropyl trimethoxy silane as a coupling agent [35,36,37,38,39,40]. However, this approach requires the chemical modification of the graphite nanoparticles with metals through the use of solvents or chemical coupling agents. Furthermore, the amount of the modified nanoparticles produced using this method is still very low; therefore, at the moment, it is not possible to treat large pieces of textile materials with this procedure. Although this chemical approach provides a good increase in the transport properties of the cotton, in our opinion, it could present some disadvantages such as high costs of the used materials (RGO, Ag, and Cu), use of potentially toxic chemical agents, limited amount of the produced nanoparticles, high process times, and low technological transferring. In order to overcome these limits, in this paper, an engineered and easily scalable process is proposed. The developed method provides prompt impregnation of the fabrics with graphite nanomaterial stacks–acetone dispersions, produced by using an economical and commercially sourced expandable graphite. This innovative approach avoids pre-coating of the fibers, therefore reducing costs and processing times. In order to verify the suitability of this method for both natural and synthetic fabrics, cotton and Lycra^®^, two widespread materials for clothing, were tested in this work. The effect of the dimensions of the produced graphite stacks–acetone-based suspensions on the electrical and thermal conductivity of both textiles was analyzed and a proper theoretical relationship between the two transport properties was successfully identified. Finally, different theoretical literature models were employed and compared with the experimental data in order to predict the transport properties.

## 2. Materials and Methods

### 2.1. Materials

Expandable graphite (EG 3772) intercalated with a mixture of sulfuric and nitric acid supplied by Anthracite Industries was employed to obtain expanded graphite (EG) flakes by means of rapid heating at 700 °C for 2 min, according to a method developed in previous works [41,42]. The EG was characterized by a specific volume of about 250 cm^3^/g and a carbon content of about 99.5%.

The textile samples were chosen between two typologies of textiles: cotton and Lycra^®^, as specimens of natural and artificial materials, respectively. Specific details about the chosen fabrics are reported in the following sections.

### 2.2. Production of Graphite Stacks–Acetone-Based Suspensions and Development of an Impregnation Method

#### 2.2.1. Production of Graphite Stacks–Acetone-Based Suspensions

One gram of EG flakes was dispersed in 500 mL of acetone (supplied by Sigma Aldrich) and sonicated in an ultrasonic bath for 5 h in order to disaggregate it into stacks. The obtained EG stacks–acetone suspension was labelled EGS. In order to verify if the size of the EG stacks in acetone could affect the transport properties of the textiles, a further treatment of EGS was implemented. In a previous paper [43], an optimized, innovative, and green process of reducing the particle size of carbon ash waste was proposed. In this paper, a proper modification of the previous method is proposed in order to reach nanometric dimensions for EG stacks–acetone suspensions. The method involved the ball milling of the EGS suspension in a horizontal oscillatory mill, MMS-Ball Mill, operating at 40 Hz for 600 h. The obtained ball-milled graphite nanomaterial stacks–acetone suspension (EGSbm) was then centrifuged for 20 min at 10,000 rpm and the supernatant was collected by obtaining a nanometric acetone–EG-based solution (EGSnm).

#### 2.2.2. Textiles Impregnation

The impregnation of cotton and Lycra^®^ samples was carried out through immersion in the developed EGS–acetone suspensions (EGS, EGSbm, and EGSnm). In order to increase the affinity between the cotton and the acetone-based suspensions, the natural fabrics were immersed in water and acetone (1:2 solution) and stirred for 30 min at room temperature. The synthetic fabrics were immersed in a 100% acetone bath and stirred for 30 min at room temperature. The impregnation of the textiles was induced by sonication. Each beaker containing textile samples immersed in EGS–acetone suspension was dipped in an ultrasonic bath and sonicated at 100% power for 1, 2, and 3 h in order to establish the optimal impregnation time.

### 2.3. Characterization Techniques

#### 2.3.1. Granulometric Analysis

The size of EGS and EGSbm was measured by Multi-Angle Light Scattering (MALS) using a CILAS 1190 particle size analyzer (Madison, WI, USA). The granulometric analysis of EGSnm was carried out using DLS (Zetasizer-Malvern, Worcestershire, UK). An optical microscope by Zeiss AxioCam (Zeiss, Oberkochen, Germany) was used for observing the fibers of the fabrics at 100× magnification after impregnation.

#### 2.3.2. Thermogravimetric Analysis

In order to estimate the weight amount of graphite stacks on the textile samples after 3 h of impregnation, the untreated and treated fabric samples were analyzed by TGA (Netzsch STA 409, NETZSCH-Gerätebau GmbH, Selb, Germany) by heating the samples from 25 to 900 °C at 10 °C/min in air atmosphere. Three replicates were performed for each sample.

#### 2.3.3. Electrical Conductivity

The electric resistance (*R*) of untreated and treated samples was measured with a PAS-853B ohmmeter (Prostat Corporation, Glendale Heights, IL, USA). The electric resistivity (*ρ*) was estimated according to Equation (1):

(1) $$\rho =6.9\;\frac{R}{s}$$

where 6.9 is an instrumental parameter related to the surface area of the instrument, expressed in [m^2^], and *s* is the thickness of the sample.

As shown in Equation (2), the electrical conductivity (*σ*) was calculated as follows:

(2) $$\sigma =\frac{1}{\rho }$$

At least five tests for all samples were performed and deviation standard was calculated according to the obtained experimental results.

#### 2.3.4. Thermal Conductivity

The thermal conductivity of the untreated and treated fabrics was calculated using DSC (Mettler Toledo 620, Greifensee, Switzerland). First, indium (melting point: 156.6 °C), chosen as sensor material, was heated at a rate of 10 °C/min with a nitrogen flow rate of 60 mL/min. Then, 12.7 mg of indium was placed into a 5 mm aluminum crucible to determine the melting curve. Indium was placed on the fabric samples and then DSC measurements were run until the melting stage of the indium was reached. At least five tests for all samples were performed and deviation standard was calculated according to the obtained experimental results.

The purpose of this measurement is to determine the thermal conductivity values by the method of Flynn and Levin [44]. By taking up the slopes of the DSC curves at melting stage of the sensor material, the thermal resistance of the sample is determined by Equation (3):

(3) $$R_{s}=R^{\prime }-R$$

where *R* is the thermal resistance between calorimeter and sensor material, *R*’ is the thermal resistance between calorimeter and sensor material with sample, and B is the heating rate. The thermal conductivity is determined by Equation (4):

(4) $$k=\frac{L}{A\;\left(R^{\prime }-R\right)}=\frac{L}{A\;R_{s}}$$

where *L* is the sample height and *A* is the contact area between sample and sensor material.

### 2.4. Mathematical Models for the Prediction of Thermal Conductivity

To estimate the thermal conductivity of the fabrics, the hierarchical structure of the fabrics must first be considered. Each fabric is composed of woven yarns, which are made by a combination of single filaments.

The prediction of thermal conductivity through mathematical models plays a key role in the production of fabrics with high thermal comfort [45]. Several models were previously studied for the estimation of the thermal conductivity of multiphase materials, which can be applied to untreated or treated textiles. For example, the calculation of the equivalent thermal conductivity of two- and three-dimensional orthogonally fiber-reinforced composites in a one-dimensional heat flow model was investigated by Tai [46], who reported a high dependence of the thermal conductivity on the volume fraction of the fabric. Krach and Advani [47] proposed a numerical system of a unit cell that is able to predict the effect of the void volume and shape on the actual conductivity of a 3-phase unidirectional composite.

Another method to predict the thermal conductivity was investigated by Militky [48], using the plain weave cell model.

The thermal conductivity of the fabrics can be estimated by different analytical models, accounting for the biphasic composition of fabrics, which are made by filaments (treated or untreated) and air. By defining KSF as the thermal conductivity of the single filament, *K_a_* as the thermal conductivity of air, and *P_o_* as the void fraction, a parallel or series model can be applied:

(5) $$K_{p}=P_{O}K_{a}+\left(1-P_{O}\right)K_{SF}$$

(6) $$K_{s}=\frac{K_{a}K_{SF}}{P_{O}K_{a}+\left(1-P_{O}\right)K_{SF}}$$

A useful analytical model was introduced by Hashin–Shtrikman [49], providing an upper limit (*K_hh_*) and a lower limit (*K_hd_*) of thermal conductivity:

(7) $$K_{hd}=\;K_{a}+\;\frac{\left(1-P_{O}\right)}{\left[\frac{1}{K_{SF}-K_{a}}+\;\frac{P_{O}}{3K_{a}}\right]}$$

(8) $$K_{hh}=K_{SF}+\frac{P_{O}}{\left[\frac{1}{K_{a}-K_{SF}}+\frac{1-P_{O}}{3K_{SF}}\right]}$$

In addition, in [50], a linear combination of series and parallels was used to estimate the thermal conductivity of fabrics:

(9) $$K_{b}=K_{SF}+\frac{K_{a}-K_{SF}}{1+\frac{1-P_{O}}{P_{O}}\left[1+z\frac{K_{a}-K_{SF}}{K_{a}+K_{SF}}\right]}$$

with *z* = 1 if all fibers are transversal to the direction of heat flow, *z* = 2/3 for random fiber orientation, and *z* = 5/6 for half of random fibers and the other half perpendicular to the direction of heat flow.

The porosity of the fabric was estimated starting with its definition:

(10) $$P_{F}=1-\rho_{W}/\rho_{F}$$

where *ρ_F_* is the density of the single filaments, and the fabric density ($\rho_{W}$) can be calculated as:

(11) $$\rho_{W}=\frac{W_{P}}{t_{W}}$$

with *W_P_* being the planar weight of the fabric, and *t_W_* its thickness.

If the yarns have negligible variations of dimensions inside the fabric, it can be shown that the combination of Equations (10) and (11) provides for the porosity of the fabric [51]:

(12) $$PF=1-\frac{\left[D_{c}T_{c}+D_{M}T_{m}\right]}{525\;10^{3}\;\rho_{F}t_{W}}$$

where $D_{C}$ and $D_{M}$ are the texture of weft and warp, respectively; and *T_C_* and *T_M_* are the corresponding textures.

For the estimation of the thermal conductivity of single filaments, *K_SF_*, used in Equations (9) and (13), the upper Hashin–Shtrikman model [50] was applied:

(13) $$K_{hh,SF}=K_{EG}+\frac{\left(1-\phi_{EG}\right)}{\left[\frac{1}{K_{y}-K_{EG}}+\frac{\phi_{EG}}{3K_{EG}}\right]}$$

in which *K_EG_* and *K_y_* are the thermal conductivities of EG and matrix (cotton or Lycra) and *ϕ_EG_* is the volume fraction of EG. Therefore, for untreated fabrics, *K_hh,SF_* reduces to *K_y_*.

## 3. Results and Discussion

The expanded graphite (EG), produced by expandable graphite, has already been characterized by authors in previous works [41,42].

**Figure 1:**
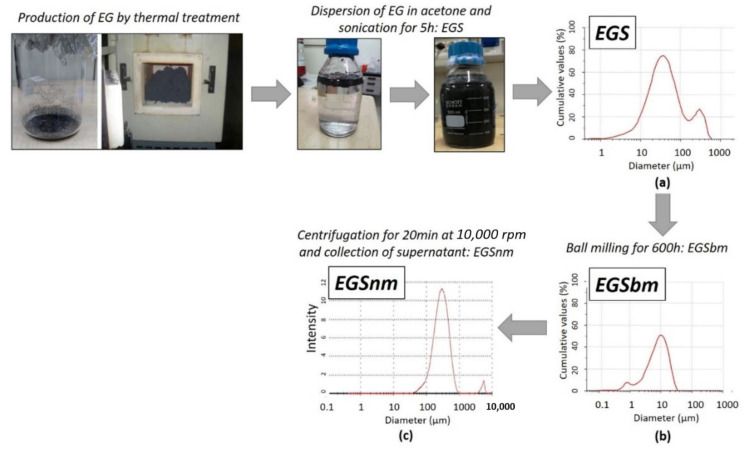
Production method of EGS-based suspensions and granulometric analysis of each one. (**a**) EGS is the graphite stacks–acetone-based suspension; (**b**) EGSbm is the ball-milled micrometric graphite stacks–acetone-based suspension; (**c**) EGSnm is the nanometric graphite stacks–acetone-based suspension.

In this study, to reduce the granulometric size of the EG previously dispersed in acetone, a proper experimental procedure was constructed. According to the method described above [43], the EGS suspension was initially subjected to ball milling. The reduction in size was then monitored by granulometric analysis carried out over time, up to 600 h. As reported in Figure 1, a marked decrease in the mean diameter of the particles was reached: the mean diameter of the EG particles decreased from 83.56 ± 1.20 µm (Figure 1a) to 10.23 ± 0.42 µm after the ball milling treatment. The micrometric graphite stacks–acetone-based suspensions obtained by the ball milling treatment were called EGSbm (Figure 1b). To reach the nanometric scale, a further treatment was employed by centrifugation of the EGSbm suspensions and by collecting the supernatant. By means of DLS analysis, it was possible to evaluate the size of the supernatant, recording values around 300 nm for the smallest particles, dispersed in acetone. The latter nanometric graphite stacks–acetone-based suspensions were called EGSnm (Figure 1c).

**Figure 2:**
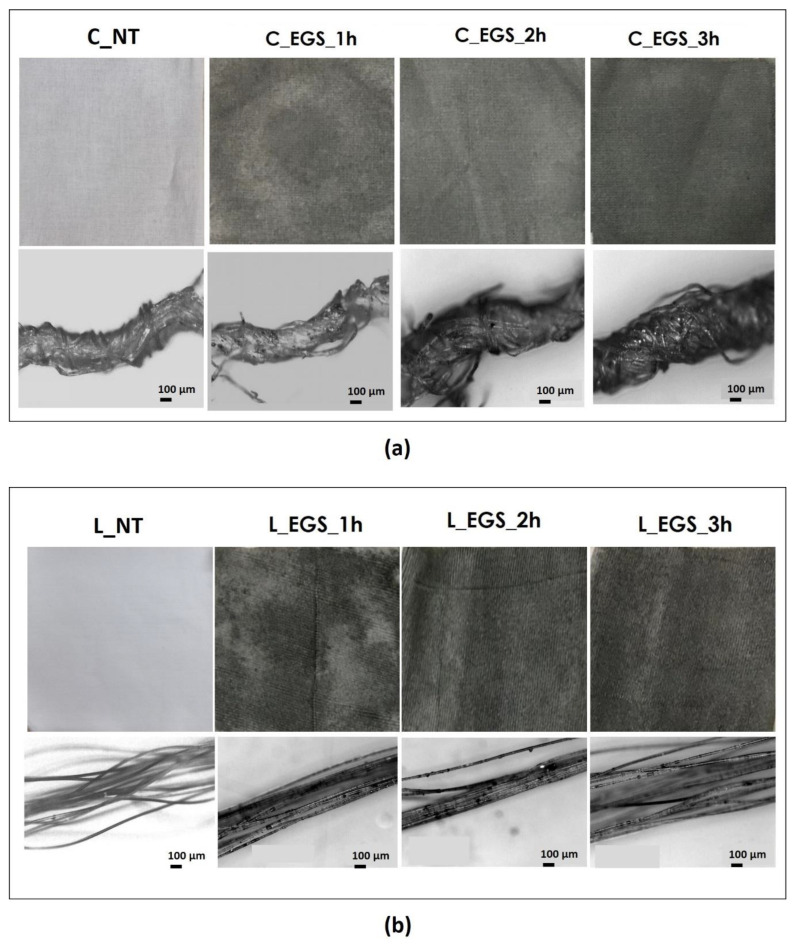
(**a**) Pictures of cotton (labelled as C) and (**b**) Lycra^®^ (labelled as L) fabrics treated with EGS–acetone suspension (labelled as EGS) at different times (labelled as 1, 2, and 3 h) (**top**) and relative micrographs of the yarns, at 100× magnification (**bottom**).

To select the optimal time of the impregnation process, the cotton and Lycra^®^ fabrics were initially impregnated for 1, 2, and 3 h using each of the acetone-based formulations produced (EGS, EGSbm, and EGSnm). Figure 2a,b shows cotton and Lycra^®^ fabrics before and after treatments, respectively. The micrograph of the yarns is reported below each fabric.

**Figure 3:**
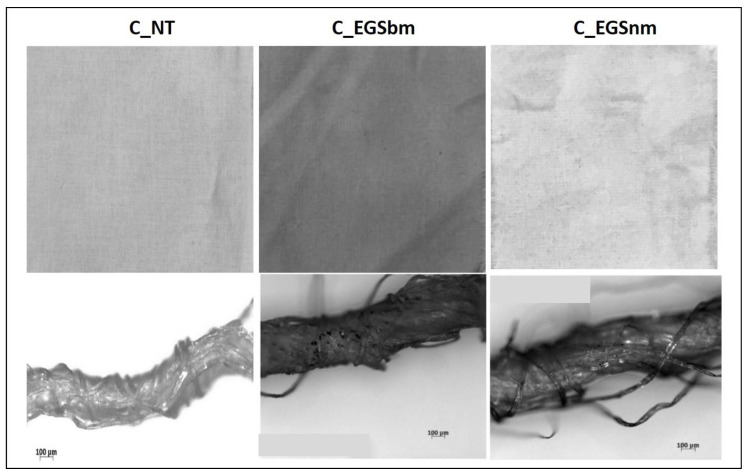
Pictures of cotton fabrics treated for 3 h with EGSbm– and EGSnm–acetone suspensions (**top**) and respective micrographs of the yarns, at 100× magnification (**bottom**).

The micrographs of Figure 2a (bottom) allowed us to investigate the EGS distribution inside the yarns. The appearance of the EGS particles on the surface of the single filaments, observed in the micrographs in Figure 2b (bottom), suggests that the developed approach allowed for EGS diffusion into the yarns. However, it is very difficult to quantify the effect of the duration of immersion by the micrographs. Instead, the pictures of the fabrics reported in Figure 2a,b show that after a longer period of immersion, the fabrics were characterized by a more intense black tone. This highlights a better and more homogeneous distribution of EGS after 3 h of impregnation for both cotton and Lycra^®^ fabrics. Thus, a time equal to 3 h was established as the optimum period of impregnation. For this reason, the cotton fabrics were also impregnated with micrometric and nanometric acetone-based suspensions (EGSbm and EGSnm) for 3 h (Figure 3).

This qualitative result suggests that the nanometric particles passed beyond the texture of the fabric, probably due to their smaller size. This indicates that the present impregnation method, which is only based on a physical interaction between the fabrics and the graphite stacks, is not suitable to be used with nanometric particles.

**Figure 4:**
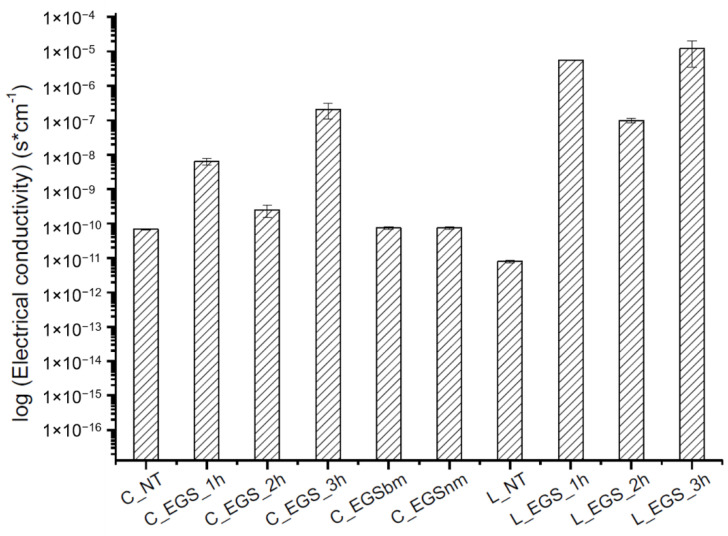
Electrical conductivity of the EGS-treated fabrics.

The electrical conductivity of all of the produced samples was measured before (cotton not treated (C_NT) and Lycra^®^ not treated (L_NT)) and after impregnation to evaluate the effect of the impregnation on the electrical properties of the fabric. As shown in Figure 4, the highest increase in electrical conductivity was reached after 3 h of treatment, while lower impregnation times did not allow optimal results, probably because of an inhomogeneous distribution of the fillers inside the yarns, as observed from the micrographs in Figure 2a,b (bottom). A reduction in the particle dimensions, obtained with ball milling, did not involve any improvement in electrical conductivity compared with untreated cotton. This result can be attributed to lower amounts of EGS in the fabric, as shown by thermogravimetric analysis (Table 1), where a lower mass residue for EGSbm-treated cotton (C_EGSbm) and EGSnm-treated cotton (C_EGSnm) was detected. The lower amount attributed to the particle dimensions indicates that the particles were too small to be locked inside the fabric (Figure 3). This result confirmed the strong difference in the color of the fabric, as previously mentioned (Figure 3, top).

Afterward, both cotton and Lycra^®^ fabrics were pressed using a hydraulic hot press, applying a pressure of 100 bar at a temperature (T) of 200 °C to obtain a preferential orientation of the graphite stacks inside the fabric. Nevertheless, the calculation of the electrical conductivity after pressure did not show any significant change; therefore, pressing of fabrics was not considered in further analyses.

The results of the electrical conductivity tests allowed discarding of the impregnation with smaller particle-based suspensions (EGSbm and EGSnm).

**Figure 5:**
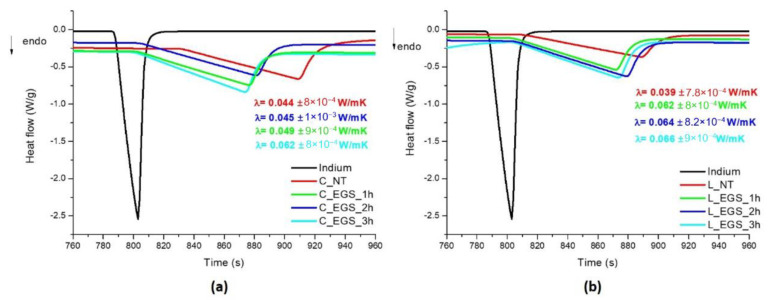
DSC curves for evaluation of the thermal conductivity of the EGS-treated (**a**) cotton and (**b**) Lycra^®^ textiles.

Therefore, thermal conductivity was calculated from DSC analyses on cotton and Lycra^®^ fabrics before and after immersion in EGS–acetone suspensions for different lengths of time. As shown in Figure 5, in accordance with electrical conductivity, the best results were, again, obtained with 3 h of impregnation.

**Figure 6:**
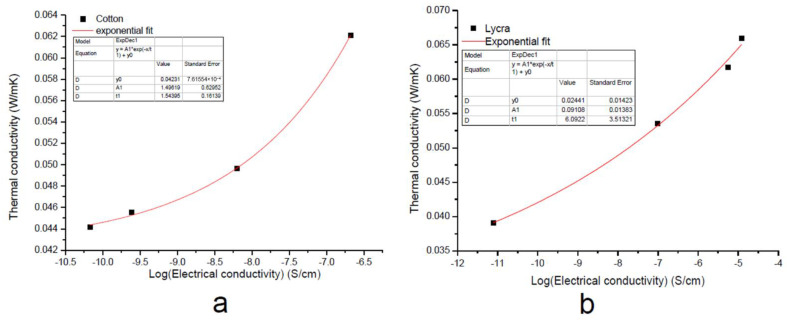
Exponential fitting of thermal conductivity of the EGS-treated (**a**) cotton and (**b**) Lycra^®^ textiles.

By plotting the thermal conductivity *λ* vs. the electrical conductivity *σ* on a semi-logarithmic scale, as reported in Figure 6, an empirical correlation was obtained, which was fitted with an exponential function of the form:

(14) $$\lambda =A1\exp\left(\frac{\log\sigma }{t1}\right)+\lambda 0$$

As reported in Figure 6, thermal conductivity increased with increasing electrical conductivity. Nevertheless, the variation in the value of the *t* parameter indicated a different growth rate between the two samples.

The thermal conductivity of the treated single filaments, estimated by the upper Hashin–Shtrikman model using the EG volume fraction of Table 1, are reported in Table 2. For the neat constituents, cotton or Lycra^®^, EG, air, and the thermal conductivity values, also reported in Table 2, were taken from [1].

After, the mathematical models of Equations (5)–(9) were used for the estimation of the thermal conductivity of the fabrics. The fabric texture parameters required for the estimation of the porosity in Equation (12) are reported in Table 3 [2].

**Figure 7:**
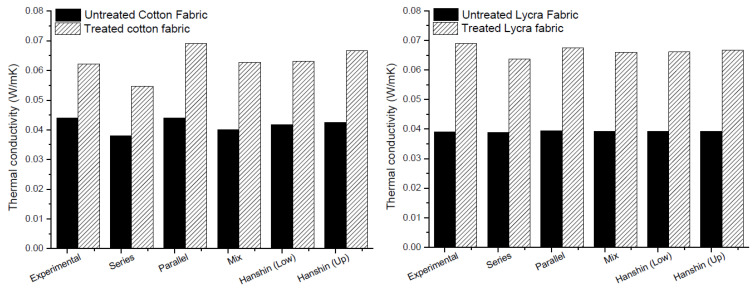
Comparison between experimental and modeling results for thermal analysis.

The thermal conductivity of the fabrics estimated in Equations (9)–(13) are compared in Figure 7 with the experimental results. As shown in Figure 7, all mathematical models fit well with the experimental results, excluding series one, which showed lower values of thermal conductivity for both fabrics. Moreover, the increase of about 50% in thermal conductivity found from experimental data was confirmed by the calculation with mathematical models for both cotton and Lycra^®^.

## 4. Conclusions

An innovative process able to reduce the micrometric size of graphite stacks to nanometric scale was proposed for the first time. The success of the method was supported by granulometric analysis, carried out during each phase of the size reduction process until reaching graphite stacks with a size of around 300 nm. As a preliminary approach, an optimized method for the impregnation of natural and artificial fabrics with graphite stacks–acetone suspensions was employed. The impregnation procedure involved a physical process, consisting of the sonication of expanded graphite–acetone-based suspensions for different lengths of time. However, since acetone-based suspensions could bleach and degrade the tissues as well as cause environmental hazards, future works will focus on a chemical modification of the graphite stacks that allows dispersion in water-based solutions.

A qualitative assessment of the impregnation was carried out by microscopic analysis, suggesting that the best impregnation was reached after 3 h. The electrical conductivity measurements confirmed that the highest increase in this transport property was reached after 3 h of treatment. This was attributed to a more homogeneous distribution of the graphite stacks inside the yarns. A reduction in the particle dimensions, obtained with the proposed ball milling process, did not produce any improvement in electrical conductivity compared with untreated cotton. This could be attributed to the dimensions of the particles, which could not be locked inside the fabric due to being too small. The results of electrical and thermal conductivity confirmed the optical microscopy results and suggested discarding of the impregnation with smaller particles at this stage. However, as previously noted, further studies are in progress to modify the graphite particles with a chemical approach, aiming to verify if the chemical modification could improve the impregnation with the nanometric particles, thus increasing the transport properties of the textiles. Furthermore, durability tests will be performed on fabrics treated with graphite stacks, with and without chemical modifications, to assess the difference between a physical and a chemical interaction of the particles within the fiber after repeated washing cycles.

An empirical correlation between thermal and electrical conductivity was also identified to predict the electrical properties of the materials, starting from the thermal experimental measurements. Finally, proper mathematical models were applied predicting the thermal conductivity of the treated textiles, obtaining a good agreement with the experimental results.

## Figures and Tables

**Table 1:** Solid residue of samples by TGA analysis and fractions of EGS for each treatment.

Sample	Solid Residue (%)	Weight Fraction (%)	Volume Fraction (%)
C_NT	4.23	−	
C_EGS1h	7.93	3.7	4.03
C_EGS2h	6.81	2.58	2.81
C_EGS3h	8.45	4.22	4.60
C_EGSbm	5.16	0.93	1.00
C_EGSnm	4.88	0.65	0.67
L_NT	10.93	−	
L_EGS1h	13.78	2.85	2.37
L_EGS2h	12.11	1.18	0.98
L_EGS3h	14.91	3.98	3.31

**Table 2:** Thermal conductivity values [1].

Material	K (W/mK)
Cotton	0.06
Air	0.024
Lycra^®^	0.065
EG	3
Treated cotton filament (Equation (13))	0.116
Lycra^®^ single filament (Equation (13))	0.138

**Table 3:** Weft and warp parameters of untreated and treated fabrics.

Material	$D_{C}\;\left[1/m\right]$	$D_{M}\;\left[1/m\right]$	$T_{C}\;\left[Tex\right]$	$T_{M}\;\left[Tex\right]$	Porosity (%)
Cotton	4000	3000	15	30	20
Lycra	5000	4000	15	30	53

## Data Availability

The data is available on reasonable request from the corresponding author.

## References

[1] Kim H., Abdala A.A., MacOsko C.W. (2010). Graphene/polymer nanocomposites. Macromolecules.

[2] Esposito Corcione C., Fasiello A., Maffezzoli A. (2007). Synthesis and characterization of boehmite reinforced epoxy nanocomposites. J. Nanostruct. Polym. Nanocompos..

[3] Esposito Corcione C., Prinari P., Cannoletta D., Mensitieri G., Maffezzoli A. (2008). Synthesis and characterization of clay-nanocomposite solvent-based polyurethane adhesives. Int. J. Adhes. Adhes..

[4] Greco A., Esposito Corcione C., Cavallo A., Maffezzoli A. (2011). Structural and thermal characterization of clay nanocomposites based on amorphous PET. J. Appl. Polym. Sci..

[5] Esposito Corcione C., Frigione M., Maffezzoli A., Malucelli G. (2008). Photo—DSC and real time—FT-IR kinetic study of a UV curable epoxy resin containing o-Boehmites. Eur. Polym. J..

[6] Esposito Corcione C., Frigione M., Acierno D. (2009). Rheological characterization of UV-curable epoxy systems: Effects of o-Boehmite nanofillers and a hyperbranched polymeric modifier. J. Appl. Polym. Sci..

[7] Esposito Corcione C., Mensitieri G., Maffezzoli A. (2009). Analysis of the structure and mass transport properties of nanocomposite polyurethane. Polym. Eng. Sci..

[8] Esposito Corcione C., Maffezzoli A. (2009). Glass transition in thermosetting clay-nanocomposite polyurethanes. Thermochim. Acta.

[9] Esposito Corcione C., Manera M.G., Maffezzoli A., Rella R. (2009). Synthesis and characterization of optically transparent epoxy matrix nanocomposites. Mater. Sci. Eng. C.

[10] Greco A., Esposito Corcione C., Strafella A., Maffezzoli A. (2010). Analysis of the structure and mass transport properties of clay nanocomposites based on amorphous PET. J. Appl. Polym. Sci..

[11] Indennidate L., Cannoletta D., Lionetto F., Greco A., Maffezzoli A. (2009). Nanofilled polyols for viscoelastic polyurethane foams. Polym. Int..

[12] Esposito Corcione C., Cavallo A., Re M., Greco A., Maffezzoli A. (2011). Evaluation of the degree of dispersion of nanofillers by mechanical, rheological and permeability analysis. Polym. Eng. Sci..

[13] Terenzi A., Vedova C., Lelli G., Mijovic J., Torre L., Valentini L., Kenny J.M. (2008). Chemorheological behaviour of double-walled carbon nanotube-epoxy nanocomposites. Compos. Sci. Technol..

[14] Rahatekar S., Zammarano M., Matko S., Koziol K., Windle A., Nyden M., Kashiwagi T., Gilman J. (2010). Effect of carbon nanotubes and montmorillonite on the flammability of epoxy nanocomposites. Polym. Degrad. Stab..

[15] Yasmin A., Daniel I.M. (2004). Mechanical and thermal properties of graphite platelet/epoxy composites. Polymer.

[16] Wang J., Wang X., Xu C., Zhang M., Shang X. (2011). Preparation of graphene/poly(vinyl alcohol) nanocomposites with enhanced mechanical properties and water resistance. Polym. Int..

[17] Nguyen D.A., Lee Y.R., Raghu A.V., Jeong H., Shin C.M., Kim B.K. (2009). Morphological and physical properties of a thermoplastic polyurethane reinforced with functionalized graphene sheet. Polym. Int..

[18] Jancar J., Jancar J. (1999). Structure-Property Relationships in Thermoplastic Matrices. Mineral Fillers in Thermoplastics I. Advances in Polymer Science.

[19] Nie Y., Hübert T. (2011). Effect of carbon nanofiber (CNF) silanization on the properties of CNF/epoxy nanocomposites. Polym. Int..

[20] Di Berardinino M.F., Pearson R.A. (1998). Fracture Behavior of Epoxy-Based, Hybrid Particulate Composites. MRS Proc..

[21] Chan C.M., Wu J., Li J.X., Cheung Y.K. (2002). Polypropylene/calcium carbonate nanocomposites. Polymer.

[22] Sumita M., Tsukumo Y., Miyasaka K., Ishikawa K. (1983). Tensile yield stress of polypropylene composites filled with ultrafine particles. J. Mater. Sci..

[23] Komarneni S. (1992). Nanocornposites. J. Mater. Chem..

[24] Kojima Y., Usuki A., Kawasumi M., Okada A., Fukushima Y., Kurauchi T., Kamigaito O. (1993). Mechanical properties of nylon 6-clay hybrid. J. Mater. Res..

[25] Giannelis E.P. (1996). Polymer Layered Silicate Nanocomposites. Adv. Mater..

[26] Chen C., Curliss D. (2001). Resin matrix composites: Organoclay-aerospace nanocomposites, Part II. Sampe J..

[27] Wang Z., Massan J., Pinnavia T.J., Pinnavia T.J., Beall G.W. (2000). Epoxy-clay nanocomposite. Polymer—Clay Nanocomposite.

[28] Yasmin A., Luo J.J., Abot J.L., Daniel I.M. (2006). Mechanical and thermal behavior of clay/epoxy nanocomposites. Compos. Sci. Technol..

[29] Abot J.L., Yasmin A., Daniel I.M. (2003). Mechanical and thermoviscoelastic behavior of clay/epoxy nanocomposites. Mater. Res. Soc. Symp. Proc..

[30] Yasmin A., Abot J.L., Daniel I.M. Processing of nanoclay/epoxy composites with a three-roll mill. Proceedings of the Materials Research Society Symposium.

[31] Bhattacharjee S., Macintyre C.R., Wen X., Bahl P., Kumar U., Chughtai A.A., Joshi R. (2020). Nanoparticles incorporated graphene-based durable cotton fabrics. Carbon.

[32] Wang Y., Hao J., Huang Z., Zheng G., Dai K., Liu C., Shen C. (2018). Flexible electrically resistive-type strain sensors based on reduced graphene oxide-decorated electrospun polymer fibrous mats for human motion monitoring. Carbon.

[33] Bhattacharjee S., Joshi R.K., Chughtai A.A., Macintyre C.R. (2019). Graphene Modified Multifunctional Personal Protective Clothing. Adv. Mater. Interfaces.

[34] Huang G., Hou C., Shao Y., Zhu B., Jia B., Wang H., Zhang Q., Li Y. (2015). High-performance all-solid-state yarn supercapacitors based on porous graphene ribbons. Nano Energy.

[35] Kowalczyk D., Fortuniak W., Mizerska U., Kaminska I., Makowski T., Brzezinski S., Piorkowska E. (2017). Modification of cotton fabric with graphene and reduced graphene oxide using sol–gel method. Cellulose.

[36] Zhao J., Deng B., Lv M., Li J., Zhang Y., Jiang H., Peng C., Li J., Shi J., Huang Q. (2013). Graphene Oxide-Based Antibacterial Cotton Fabrics. Adv. Healthc. Mater..

[37] Cai G., Xu Z., Yang M., Tang B., Wang X. (2017). Functionalization of cotton fabrics through thermal reduction of graphene oxide. Appl. Surf. Sci..

[38] Stan M., Badea M.A., Pircalabioru G.G., Chifiriuc M.C., Diamandescu L., Dumitrescu I., Trica B., Lambert C., Dinischiotu A. (2019). Designing cotton fibers impregnated with photocatalytic graphene oxide/Fe, N-doped TiO2 particles as prospective industrial self-cleaning and biocompatible textiles. Mater. Sci. Eng. C.

[39] Lu Y., Xiao X., Liu Y., Wang J., Qi S., Huan C., Liu H., Zhu Y., Xu G. (2020). Achieving multifunctional smart textile with long afterglow and thermo-regulation via coaxial electrospinning. J. Alloys Compd..

[40] Pan N., Liu Y., Ren X., Huang T.-S. (2018). Fabrication of cotton fabrics through in-situ reduction of polymeric N-halamine modified graphene oxide with enhanced ultraviolet-blocking, self-cleaning, and highly efficient, and monitorable antibacterial properties. Colloids Surf. A Physicochem. Eng. Asp..

[41] Corcione C.E., Freuli F., Maffezzoli A. (2013). The Aspect Ratio of Epoxy Matrix Nanocomposites Reinforced with Graphene Stacks. Polym. Eng. Sci..

[42] Bourdo S.E., Warford B.A., Viswanathan T. (2013). Electrical and thermal properties of graphite/polyaniline composites. Polym. Eng. Sci..

[43] Striani R., Stasi E., Giuri A., Seiti M., Ferraris E., Esposito Corcione C. (2021). Development of an innovative and green method to obtain nanoparticles from carbon-based waste ashes. Nanomaterials.

[44] Flynn J.H., Levin D.M.A. (1988). A method for the determination of thermal conductivity of sheet materials by DSC. Thermochim. Acta.

[45] Militký J., Křemenáková D.A. Simple Methods for Prediction of Textile Fabrics Thermal Conductivity—Paper number: PJ4. Proceedings of the 5th International Conference on Heat Transfer, Fluid Mechanics and Thermodynamics.

[46] Tai H. (1996). Equivalent Thermal Conductivity of Two- and Three-Dimensional Orthogonally Fiber-Reinforced Composites in One-Dimensional Heat Flow. J. Compos. Technol. Res..

[47] Krach A., Advani S. (1996). Influence of void shape, void volume and matrix anisotropy on effective thermal conductivity of a three-phase composite. J. Compos. Mater..

[48] Militký J., Křemenáková D. Prediction of textile fabric thermal conductivity. Proceedings of the 5th HEFAT Conference.

[49] Hashin Z. (1962). The elastic moduli of heterogeneous materials. J. Appl. Mech..

[50] Al Sulaiman F.A., Al-Nassar Y.N., Mokheimer E.M.A. (2006). Numerical prediction of thermal conductivity of fibres. Heat Mass Transf..

[51] Militký J., Trávníčková M., Bajzík V. (1999). Air Permeability and Light Transmission of Weaves, Fibres and Textiles. Int. J. Cloth. Sci. Technol..

